# Think Addison’s Disease: Disseminated Tuberculosis Presenting With Adrenal Crisis in a Young Male Patient

**DOI:** 10.7759/cureus.94091

**Published:** 2025-10-08

**Authors:** Hassan Mohamed, Ahmed M Attia, Harrie Toms John, Ahmed Owies, Matt Varrier

**Affiliations:** 1 Intensive Care, Epsom and St Helier University Hospitals NHS Trust, London, GBR; 2 Intensive Care, Manchester Royal Infirmary, London, GBR

**Keywords:** addison’s disease, adrenal crisis, disseminated tuberculosis, intensive care, primary adrenal insufficiency

## Abstract

A 42-year-old Indian man presented to the emergency department with hypotensive collapse, initially presumed to be septic shock. He had a background of progressive weight loss, fatigue, anaemia, and a strict vegan and lactose-free diet requiring vitamin B12 supplementation in the past. Despite fluid resuscitation and broad-spectrum antibiotics, his shock persisted. Profound hyponatremia (Na 106 mmol/L) with hyperkalaemia (K 5.6 mmol/L), diffuse skin hyperpigmentation, and lab tests showed low cortisol and clinical features of adrenal insufficiency, supported by imaging and culture-confirmed tuberculosis (TB). Further evaluation revealed disseminated TB as the underlying cause. CT imaging showed bilateral adrenal enlargement (adrenalitis) without destruction, along with cavitary pulmonary lesions, a “tree-in-bud” pattern in the lungs, lymphadenopathy, and TB foci in the bursa and scrotum. TB infection was confirmed with a positive culture. The patient was treated with high-dose intravenous hydrocortisone and fluid/electrolyte support, followed by lifelong glucocorticoid and mineralocorticoid replacement and a full course of anti-tubercular therapy. He gradually stabilised, with resolution of hypotension and electrolyte imbalances, and showed clinical improvement on follow-up. This case highlights the importance of considering adrenal crisis in refractory hypotension, particularly in younger patients, and underscores the rare presentation of early adrenal TB with preserved gland shape, demonstrating how disseminated TB, facilitated by diet-related malnutrition, can present as Addison’s disease with characteristic adrenal imaging findings and increased severity.

## Introduction

Primary adrenal insufficiency (Addison’s disease) is a potentially fatal disorder that often goes unrecognised in its early stages because the symptoms are vague and nonspecific [1]. In younger patients, adrenal crisis is frequently mistaken for more common conditions such as sepsis, which can result in dangerous delays in diagnosis [2]. Evidence shows that 40-50% of patients experience symptoms for more than six months before receiving a diagnosis, and in up to two-thirds, the disease is only identified during hospitalisation for an adrenal crisis [3]. These crises carry substantial risk, with a mortality rate of approximately 0.5% per episode, and fatal outcomes have been documented even in previously healthy young individuals when the condition was missed [3].

Historically, tuberculosis (TB) of the adrenal glands, first described by Thomas Addison in 1855, was the leading cause of Addison’s disease [1]. Today, with falling TB prevalence worldwide, autoimmune adrenalitis has become the most common cause in many regions [3]. Nonetheless, adrenal TB remains a key consideration in TB-endemic settings and among immunocompromised individuals [1]. Reactivation of latent TB is often triggered by immune suppression, with recognised risk factors including HIV infection, diabetes, malnutrition, and other states of immunosuppression [4].

Chronic nutritional deficiencies further weaken host immunity. Diets that exclude animal products, such as strict vegan or some vegetarian patterns, can lead to protein-calorie malnutrition and micronutrient deficits (notably vitamin B12 and vitamin D), impairing cell-mediated immune responses [5]. Epidemiological studies have even suggested a link between vegetarian diets and increased TB susceptibility: one study reported an 8.5-fold higher risk of TB among lacto-vegetarians compared to meat-eaters, likely reflecting reduced immunocompetence [5].

This case is presented for its educational importance: it highlights a delayed diagnosis of Addison’s disease in a young adult, precipitated by disseminated TB in the setting of nutritional risk factors. It also emphasises the distinctive imaging features of early adrenal TB, notably adrenal enlargement without calcification, and underscores the need for heightened clinician awareness of this treatable cause of adrenal failure.

## Case presentation

A 42-year-old man of Indian origin was brought to the emergency department after suddenly collapsing at his general practitioner’s clinic. On arrival, he was profoundly hypotensive with a blood pressure of 70/40 mmHg, tachycardic at 110 bpm, and drowsy with a Glasgow Coma Scale score of 13 (eyes 3, verbal 4, motor 6). He appeared cachectic, with diffuse bronzed hyperpigmentation involving sun-exposed areas, palmar creases, knuckles, and the buccal mucosa. His extremities were cool with delayed capillary refill, and he was in overt circulatory shock. No focal signs of infection were detected, heart sounds were rapid but normal, jugular venous pressure was low, and the abdomen was soft without organomegaly. Mild generalised muscle weakness was present, though no focal neurological deficits were observed.

The patient’s history revealed intermittent night sweats, episodic low-grade fevers, and chronic fatigue for over two years. Friends had noticed his progressive weight loss in late 2023, which was later corroborated by serial photographs showing facial fat loss and emerging cachexia. He had lost more than 20 kilograms over the past three months alone. In March 2024, he was hospitalised with community-acquired pneumonia and received intravenous antibiotics without adrenal testing. One year later, in March 2025, he required a second admission for pneumonia, during which persistent anaemia (Hb 70-80 g/L) was treated with ferrous fumarate and intramuscular vitamin B12 injections. Despite this, he continued to complain of anorexia, episodic dizziness, alternating dry and productive cough, and drenching night sweats. On April 27, 2025, one day before presentation, he experienced two brief blackout episodes at home while standing. A summary of the clinical timeline is provided in Table 1.

**Table 1:** Timeline of key events in patient's history until admission

Timeline of Key Events	Clinical Details
≥ 2 years before ED visit	Intermittent night sweats, episodic low-grade fevers, and chronic fatigue.
December 2023	Friends noticed progressive weight loss; review of serial photos later showed loss of facial fat and emerging cachexia.
March 2024	Hospitalised for community-acquired pneumonia (CAP); received IV antibiotics, discharged without adrenal testing.
March 2025	Second CAP admission; persistent anaemia (Hb 70-80 g/L) treated with ferrous fumarate and intramuscular vitamin B₁₂; no gastrointestinal bleeding ever documented.
April 2025	Alternating dry and productive cough; drenching night sweats every other night; episodic dizziness and anorexia.
27 Apr 2025 (day -1)	Two brief blackout episodes at home while standing.
28 Apr 2025 (ED day 0)	Collapsed with dyspnea; brought by ambulance.

His past medical history included iron-deficiency anaemia, strict adherence to a vegan and lactose-free diet, and prior vitamin B12 deficiency requiring intramuscular supplementation. He had undergone laparoscopic cholecystectomy in 2012 and denied corticosteroid use. Social history revealed a 20-year smoking history (four cigarettes daily) and moderate alcohol intake (2-3 pints on five days a week until early 2025, now weekends only). There was no known HIV or other immunosuppressive condition. Family history was significant: his father had died of “bronchopneumonia” at a young age, raising suspicion of undiagnosed TB, while his mother was successfully treated for pneumonia in 2023.

Initial laboratory investigations on admission demonstrated severe hyponatremia (106 mmol/L), mild hyperkalemia (5.6 mmol/L), hypoglycemia (3.1 mmol/L), and normocytic anaemia (Hb 82 g/L on admission, falling to a nadir of 69 g/L during hospitalisation; prior Hb 70-80 g/L in March 2025). Ferritin was elevated at 3102 µg/L, C-reactive protein 67 mg/L, and random cortisol was 102 nmol/L, supporting primary adrenal insufficiency. ACTH was not measured during admission because the patient had already been started on immunosuppressive therapy, which would confound interpretation. Arterial blood gases showed compensated metabolic acidosis (pH 7.42, HCO₃⁻ 20 mEq/L, base excess -6.3). Renal function was preserved. Inflammatory markers included WBC 3.5×10^9/L with normal neutrophils. The evolution of his blood results throughout admission and at discharge is summarised in Table 2.

**Table 2:** Evolution of blood results Hb: haemoglobin; WBC: white blood cell count; CRP: C-reactive protein; eGFR: estimated glomerular filtration rate; HCO₃: bicarbonate; ALT: alanine aminotransferase; LDH: lactate dehydrogenase.

Parameter	On admission	During admission	At discharge	Reference values
Haemoglobin (Hb, g/L)	82	98	88	120–160
White blood cell count (WBC, ×10⁹/L)	7.0	11.5	9.6	4.0–11.0
Platelet count (×10⁹/L)	171	216	188	150–400
C-reactive protein (CRP, mg/L)	67.0	38.9	37.1	<5
Sodium (mmol/L)	106	133	133	135–145
Potassium (mmol/L)	5.6	4.4	4.0	3.5–5.0
Urea (mmol/L)	–	4.4	4.0	2.5–7.0
Creatinine (µmol/L)	102	59	54	45–90
Estimated glomerular filtration rate (eGFR, mL/min/1.73m²)	–	>90	>90	>90
Bicarbonate (HCO₃, mmol/L)	20	23	–	22–29
Base excess	–4.9	–2.7	–	–2 to +2
Cortisol (nmol/L)	102	566	333	150–650 (morning)
Total bilirubin (µmol/L)	61	37	24	<21
Alanine aminotransferase (ALT, U/L)	–	45	37	<40
Albumin (g/L)	–	33	33	35–50
Calcium (mmol/L)	–	2.24	2.31	2.15–2.55
Phosphate (mmol/L)	–	1.26	1.25	0.8–1.5
Lactate dehydrogenase (LDH, U/L)	–	261	238	135–225
Ferritin (µg/L)	–	938	566	30–400

**Figure 1:**
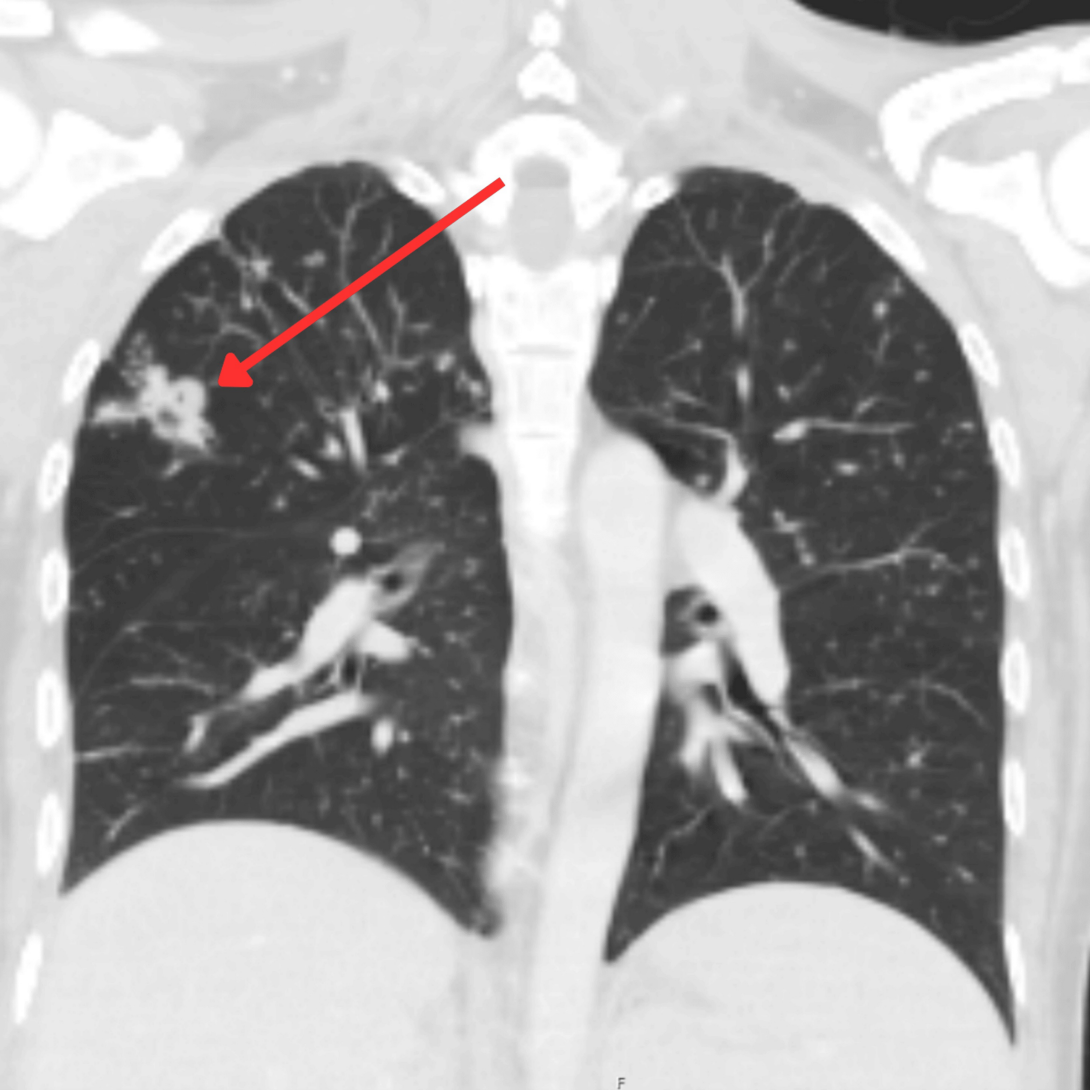
CT chest demonstrating a spiculated cavitating opacity in the right upper lobe, indicated by the red arrow.

**Figure 2:**
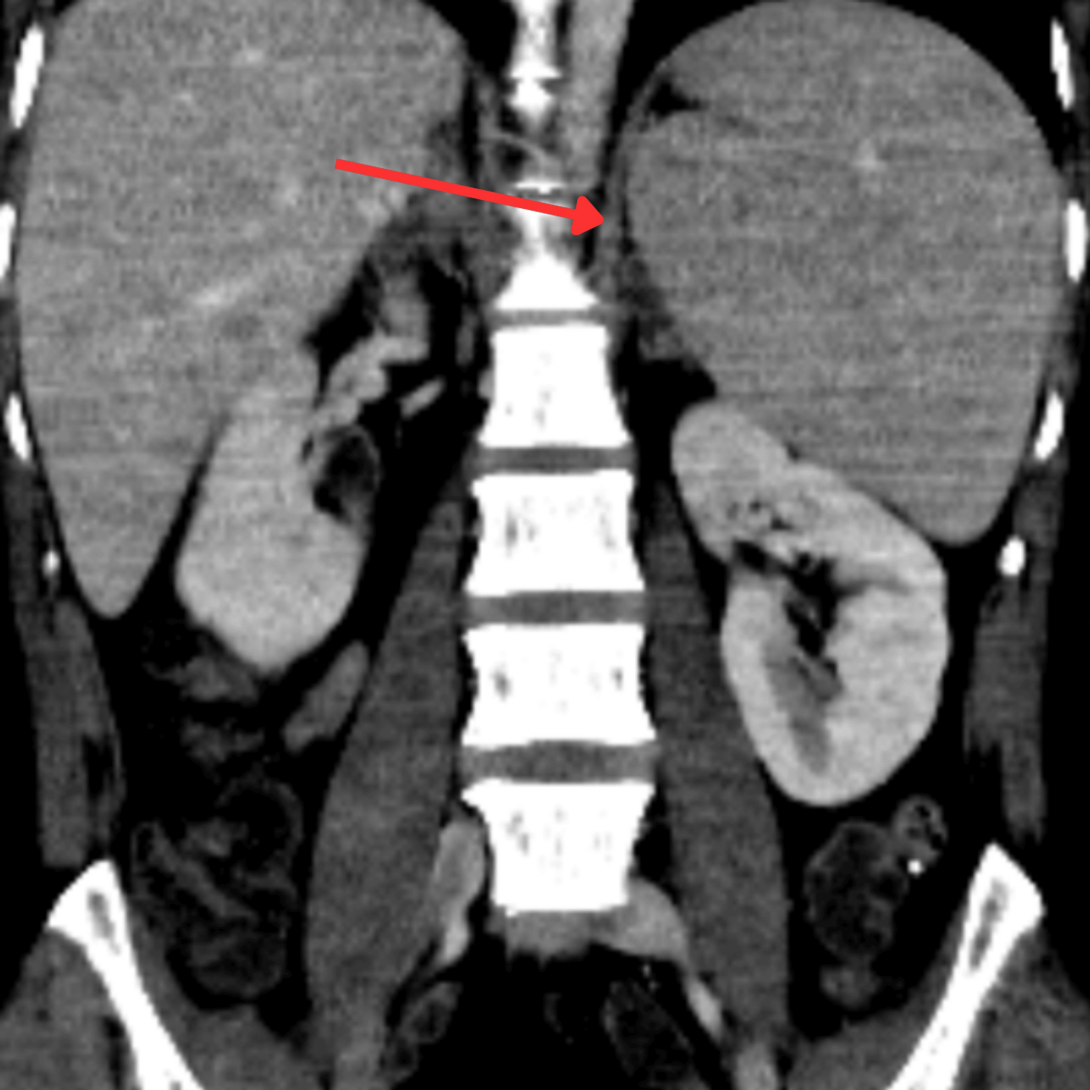
CT abdomen showing splenomegaly (red arrow), supporting disseminated tuberculosis. No focal splenic lesion is seen.

Given his refractory hypotension, septic shock was initially suspected and broad-spectrum antibiotics were commenced alongside IV fluids. Despite this, blood pressure did not respond, necessitating vasopressor support. The constellation of profound hyponatremia, hyperkalemia, hypoglycemia, and hypotension subsequently raised suspicion of adrenal insufficiency. A CT of the head, chest, abdomen, and pelvis revealed a 2.4 cm spiculated cavitating opacity in the right upper lung lobe, extensive peribronchovascular nodularity, and bilaterally abnormal adrenal glands consistent with subacute adrenalitis (Figure 1 and Figure 2). The CT abdomen also demonstrated splenomegaly (Figure 2), supporting disseminated TB involvement.

**Figure 3:**
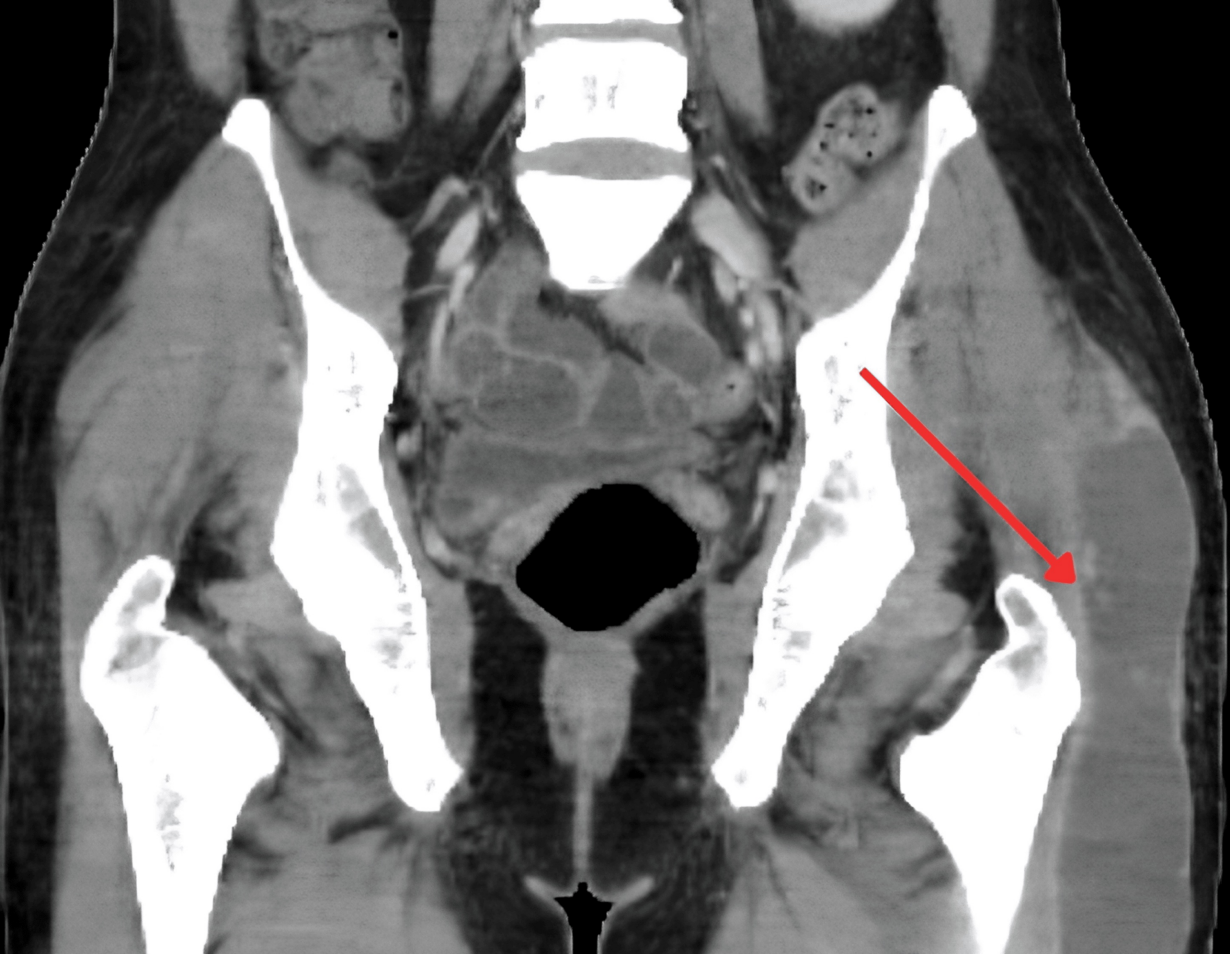
CT showing large thick-walled low-density and peripherally enhancing collection overlying the left greater trochanter, likely tuberculous bursitis. The lesion supports systemic dissemination of TB. TB: Tuberculosis

Other observations include a large, thick-walled low-density and peripherally enhancing collection overlying the left greater trochanter, likely acute trochanteric bursitis. Within the left scrotum, there is a peripherally enhancing, centrally low-density lesion which is equivocal (Figure 3).

The CT abdomen findings of splenomegaly and a large, thick-walled low-density collection overlying the left greater trochanter are consistent with musculoskeletal involvement of disseminated TB. These findings further supported hematogenous spread and systemic disease.

An urgent dose of intravenous hydrocortisone (100 mg) was administered in the emergency department, leading to marked hemodynamic improvement. Over the following 48 hours, blood pressure stabilised, vasopressors were discontinued, serum sodium rose to 128 mmol/L, and potassium normalised to 4.0 mmol/L. The diagnosis of Addisonian crisis was established, and attention turned to the underlying cause.

At this stage, the constellation of hypotension, severe hyponatremia, hyperkalemia, and low cortisol confirmed the presence of primary adrenal insufficiency. The differential diagnosis for an acute Addisonian crisis included septic shock, cardiogenic collapse, and neurogenic shock. Septic shock was initially favoured; however, the absence of a clear septic focus and refractory hypotension despite antibiotics argued against this. Cardiogenic and neurogenic causes were excluded by normal echocardiography and the absence of trauma or neurological signs.

Once adrenal insufficiency was established, the key question became its underlying cause. In a 42-year-old man, possible etiologies include autoimmune adrenalitis, infections (particularly TB in endemic populations), bilateral adrenal haemorrhage, metastatic malignancy, and less common genetic or drug-induced causes. The acute deterioration with preceding systemic symptoms argued against a slowly progressive autoimmune process. Adrenal haemorrhage was unlikely given the lack of coagulopathy or meningococcal infection, and CT imaging showed enlargement with preserved shape rather than hemorrhagic destruction. Metastatic disease was considered, especially given bilateral adrenal involvement, but the presence of pulmonary cavitation, night sweats, and positive TB cultures made disseminated TB the most unifying diagnosis. Although ACTH measurement was unavailable due to prior immunosuppressant therapy, the combination of low cortisol, characteristic clinical and imaging findings, and positive Mycobacterium tuberculosis cultures confirmed the diagnosis of primary adrenal insufficiency due to adrenal TB. Adrenal lymphoma was another theoretical consideration, though it would not explain the pulmonary lesions and typically presents with different enhancement patterns on CT.

**Figure 4:**
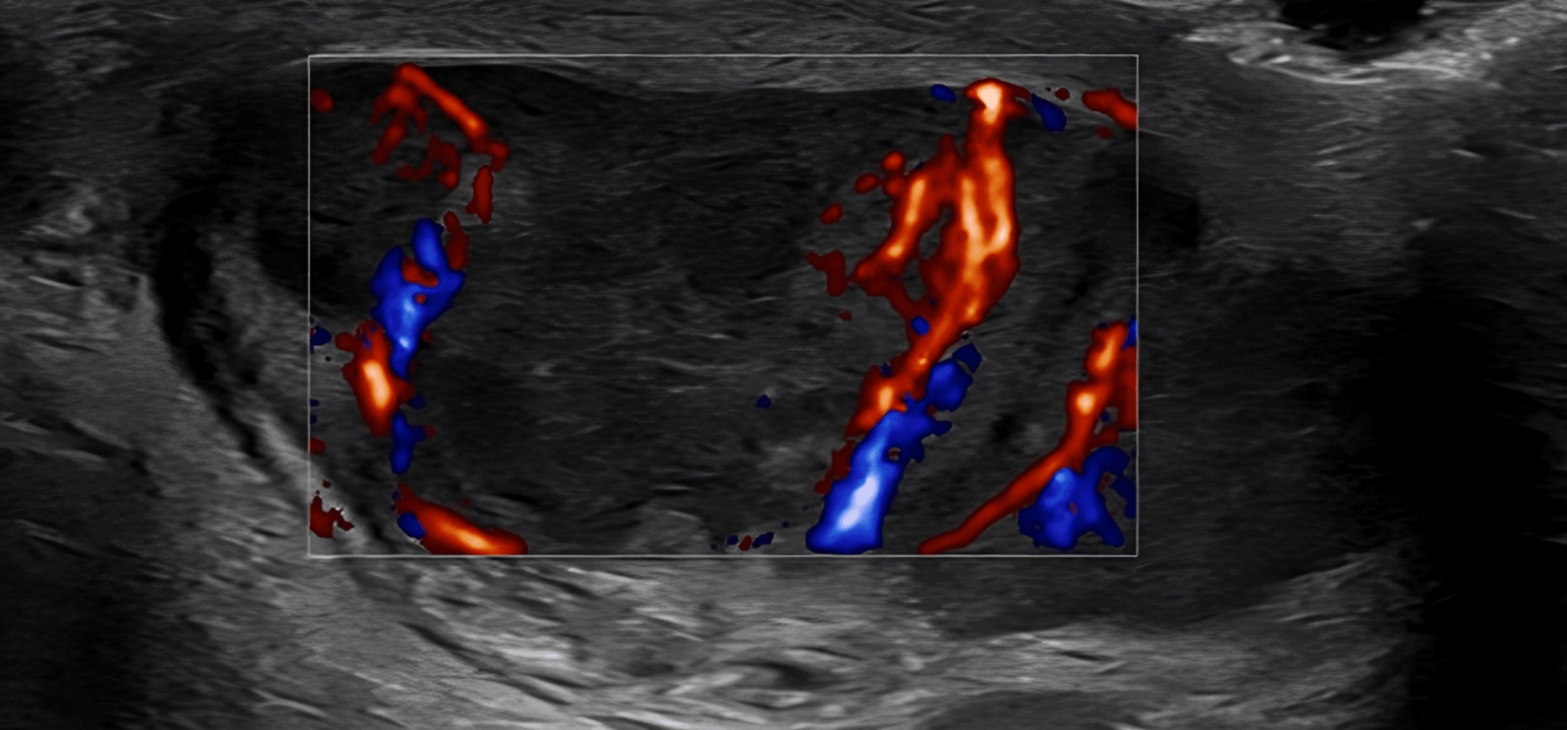
Ultrasound scrotum showing bilateral epididymo-orchitis.

**Figure 5:**
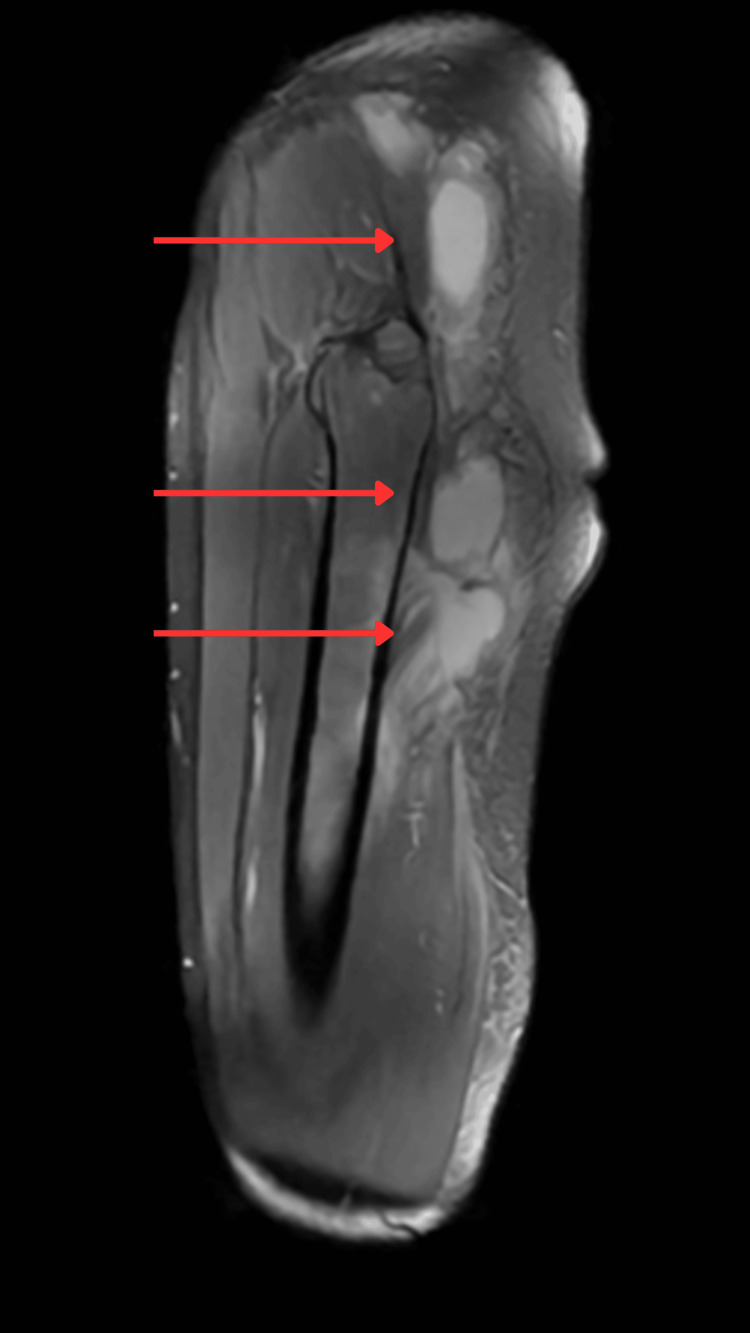
MRI left thigh showing a multilobulated abscess indicated with red arrows.

**Figure 6:**
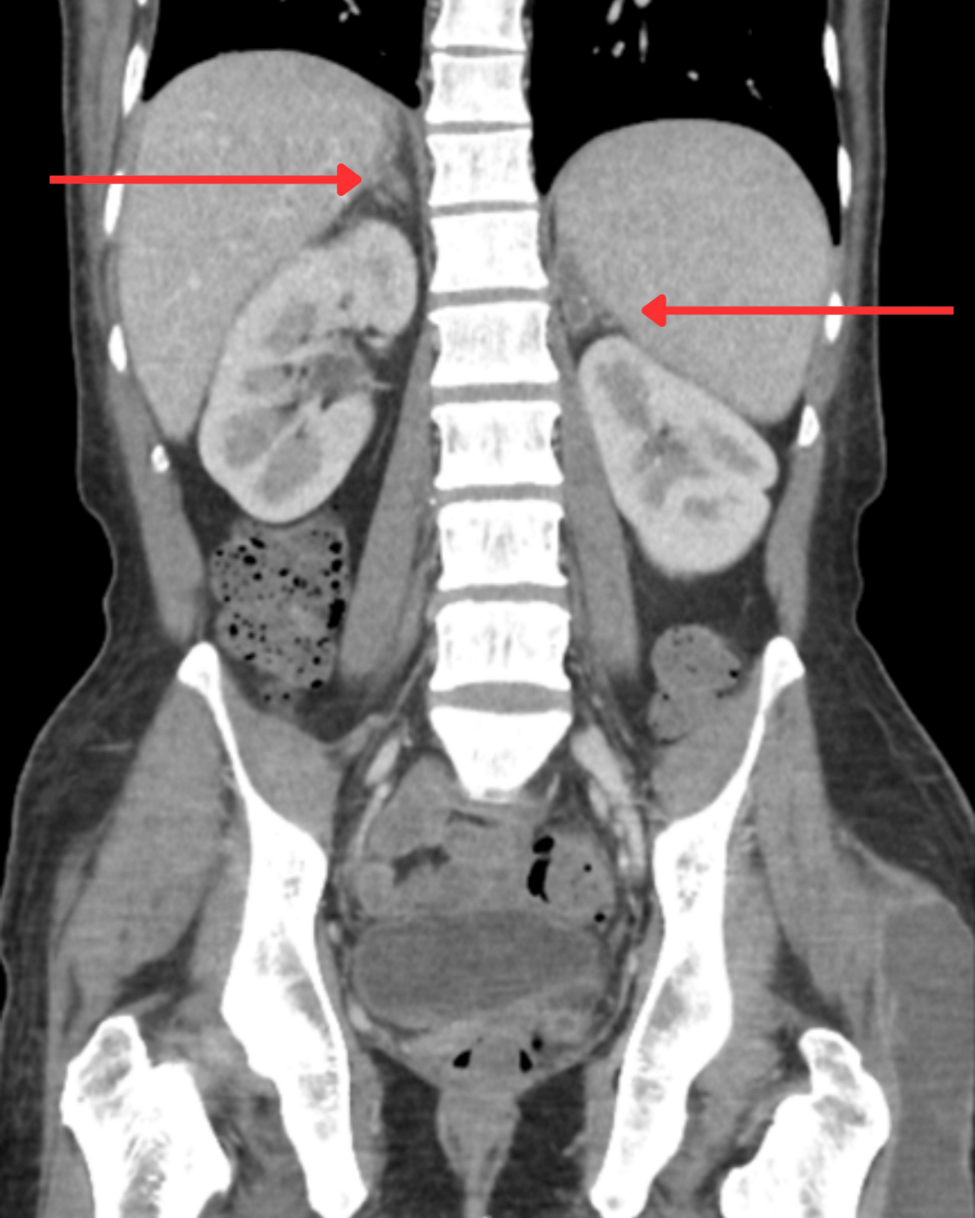
Follow-up CT adrenal glands demonstrating features of adrenal TB indicated by red arrows.

Although initial sputum smears for acid-fast bacilli were negative, cultures grew M. tuberculosis after one week of incubation. An interferon-gamma release assay (QuantiFERON-TB Gold) was not performed; however, TB was confirmed via positive cultures, which eliminated the need for IGRA testing. Urine and CSF analyses showed no TB involvement. Although ACTH measurement was unavailable due to prior immunosuppressant therapy, the combination of low cortisol, characteristic clinical and imaging findings, and positive M. tuberculosis cultures confirmed the diagnosis of primary adrenal insufficiency due to adrenal TB. Autoimmune screening for adrenalitis was negative. Further imaging revealed disseminated disease: ultrasound of the scrotum demonstrated bilateral epididymo-orchitis with probable infarction of the left testis (Figure 4), MRI of the thigh identified a multilobulated collection consistent with TB abscess (Figure 5), and follow-up CT of the adrenal glands showed peripheral enhancement with punctate calcification in the left adrenal gland, characteristic of adrenal TB (Figure 6).

**Figure 7:**
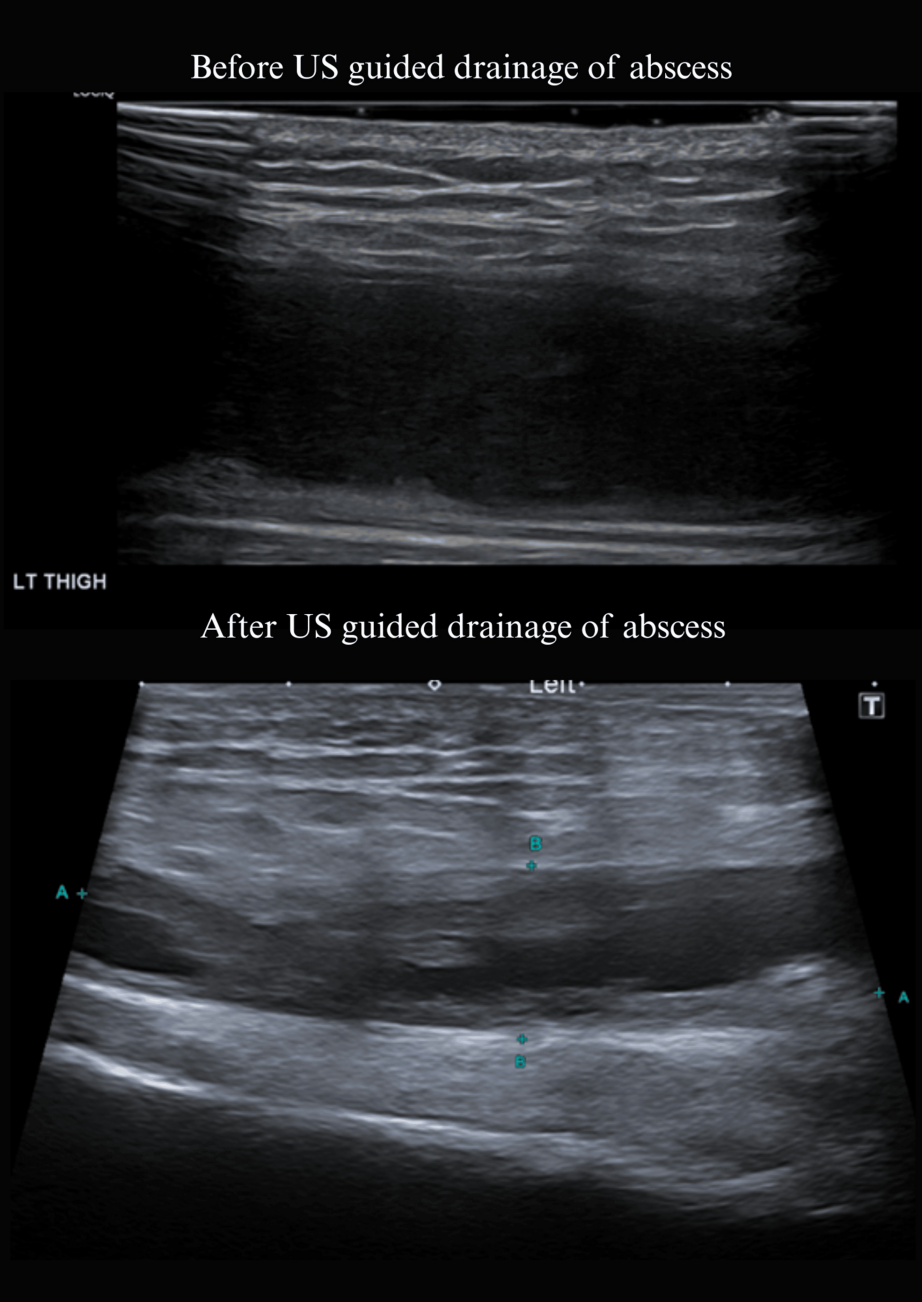
US left thigh showing before and after US-guided drainage of the abscess.

Ultrasound-guided drainage of the thigh abscess confirmed TB on culture and PCR. With these findings, a unifying diagnosis of disseminated TB with tuberculous adrenalitis was established (Figure 7).

The patient was managed in the intensive care unit with intravenous hydrocortisone (100 mg bolus, then 50 mg every six hours), IV saline for volume resuscitation, and careful correction of hyponatremia. Once stabilised, fludrocortisone (0.1 mg daily) was added to address mineralocorticoid deficiency. Empirical antibiotics were discontinued when bacterial sepsis was excluded, and he was commenced on quadruple anti-tubercular therapy (isoniazid, rifampicin, pyrazinamide, and ethambutol) with pyridoxine supplementation. Nutritional support was provided with iron, folate, vitamin B12, and vitamin D3 repletion.

By day 5 of admission, his condition had improved significantly, allowing transition to oral hydrocortisone and oral anti-tubercular therapy. He was educated on Addison’s disease management, including the importance of stress-dose steroids and medical alert identification.

After 26 days in hospital, he was discharged hemodynamically stable with oral hydrocortisone (20 mg three times daily), fludrocortisone (150 µg daily), and continuation of four-drug TB therapy. At discharge, sodium and potassium were normalised, and blood pressure was stable. On follow-up, he reported improved appetite, energy, weight gain of 2 kg, and complete resolution of cough and night sweats.

At three-month follow-up, ACTH was elevated with 309pg/mL (reference range: 7.2-63.3 pg/mL), indicating persistent primary adrenal insufficiency; ongoing replacement therapy was continued. Sputum cultures converted to negative, consistent with treatment response.

## Discussion

This case illustrates several important learning points regarding adrenal insufficiency and disseminated TB in a young adult. First, it highlights the diagnostic challenge and dangers of delayed recognition of primary adrenal insufficiency. Addison’s disease often presents with insidious, nonspecific symptoms (fatigue, weight loss, gastrointestinal complaints) that can be mistaken for more common ailments [1]. In acute settings, an Addisonian crisis may masquerade as septic shock, as seen in our patient, where the initial misdiagnosis was sepsis. Similar cases have been reported where adrenal crisis was only recognised after patients failed to respond to usual shock management [2]. Unfortunately, postponing the correct diagnosis can have dire consequences; adrenal crisis is a true endocrine emergency with an estimated fatality of about 0.5% per episode even under care [3]. Especially in young patients, clinicians may not have a high index of suspicion, leading to multiple healthcare encounters over many months before the underlying adrenal insufficiency is finally identified [3]. Our patient had nearly a year of progressive fatigue and weight loss, which, in retrospect, were red flags for adrenal insufficiency. The case of a 13-year-old girl who died from an unrecognised Addison’s disease described by Jimenez and Crossen (2023) is a poignant reminder of the stakes [3]. In that report, as in ours, key clinical clues (skin hyperpigmentation, electrolyte disturbances) were initially overlooked. Education to “think of Addison’s in unexplained hypotension and hyponatremia” is critical. Once identified, primary adrenal insufficiency can be readily treated with hormone replacement, preventing unnecessary morbidity and mortality [6]. In our case, prompt administration of IV hydrocortisone likely saved the patient’s life and led to rapid hemodynamic improvement.

Second, this case underscores TB as an important cause of Addison’s disease, particularly in endemic areas or vulnerable populations. Tuberculous adrenalitis was the leading cause of Addison’s disease in the pre-steroid era and remains a consideration worldwide when autoimmune causes are excluded [1]. TB most commonly reaches the adrenal glands via hematogenous spread from a primary focus (often the lungs or genitourinary system) [1]. In autopsy studies of TB patients, adrenal involvement has been observed in roughly 6% of cases [6]. However, clinically apparent adrenal insufficiency develops only if a majority of the adrenal cortex is destroyed; it is estimated that >90% of glandular tissue must be lost before cortisol production falls to symptomatic levels [1]. This typically corresponds to advanced, bilateral adrenal TB, usually many years after initial infection [1]. Our patient’s presentation is consistent with this timeline: as a middle-aged man from India, it is likely he was infected with M. tuberculosis in his youth, with latent TB reactivating decades later under conducive conditions. His father’s “bronchopneumonia” could very well have been TB, suggesting a source of exposure.

Interestingly, in this patient, the imaging findings showed adrenal enlargement (hypertrophy) rather than atrophy, indicating an active inflammatory phase of adrenal TB. Radiologically, tuberculous adrenal glands go through stages: in early active infection, the glands are typically enlarged with a smooth or mildly lobulated contour, due to caseous granulomatous inflammation and oedema [1]. There may be central low-density areas on CT if necrosis is present, often with a rim of peripheral enhancement after contrast injection (reflecting viable adrenal cortex and inflammatory tissue) [1]. Crucially, the normal adrenal shape can be preserved in this phase [7]. In chronic or “burnt-out” adrenal TB, the glands tend to calcify, fibrose, and shrink, sometimes becoming difficult to discern on imaging except for calcifications [1]. Calcifications are seen in up to 50% of adrenal TB cases on CT, especially in longstanding disease [7]. Our patient’s CT demonstrated bilateral bulky adrenal glands with rim enhancement and no calcifications, which is characteristic of active TB adrenalitis prior to complete gland destruction. This has practical implications: it is possible that some adrenal function was initially preserved. There are reports of partial recovery of adrenal function if anti-TB therapy is started early in the adrenalitis stage [7], though in most cases like ours, adrenal insufficiency remains permanent due to irreversible damage [1]. The differential diagnosis for bilateral adrenal masses/enlargement in a TB-endemic context includes adrenal histoplasmosis and other infections, bilateral metastases, or lymphoma. Certain radiologic clues favour TB; for example, peripheral rim enhancement with central necrosis and calcifications on CT, especially with coexisting TB elsewhere, strongly suggests adrenal TB [1]. By contrast, metastatic lesions might show irregular enhancement or larger discrete tumours, and lymphoma would usually present with lymph node involvement and a different clinical picture. In our patient, the co-occurrence of classic lung TB findings and positive microbiology clinched the diagnosis. It is worth noting that even with modern imaging, adrenal TB is often diagnosed late; one population-based study from Sweden found adrenal TB to be extremely rare but associated with high mortality, in part because it was discovered only when adrenal failure was advanced [8]. Therefore, clinicians should proactively screen for adrenal insufficiency in patients with disseminated or multifocal TB, as early intervention with corticosteroids is lifesaving.

Third, this case brings attention to the interaction between nutrition, immunity, and TB reactivation. The patient’s lifestyle as a strict vegan on a lactose-free diet likely contributed to a state of chronic malnutrition, evidenced by his weight loss, anaemia, and vitamin deficiencies. Malnutrition is well-known to modulate immune responses and is the single most important risk factor for progression of latent TB infection to active disease in many developing regions [9]. Protein-energy undernutrition and micronutrient deficiencies (such as vitamin A, D, E, B-complex, iron, zinc, and selenium) can impair both innate and adaptive immunity, reducing the host’s ability to contain M. tuberculosis [9]. In particular, cell-mediated immunity, the cornerstone of defence against TB, is hampered by nutritional deficiencies. For example, low protein intake can cause T-cell dysfunction and thymic atrophy, while deficiencies in iron and vitamin B12 can impair macrophage and neutrophil function [5]. Our patient’s longstanding B12 deficiency (requiring injections) and anaemia suggest that his diet was insufficient to sustain an effective immune response. Vitamin D status is another crucial factor; vitamin D plays a key role in macrophage activation and production of antimicrobial peptides (like cathelicidin) that kill TB bacilli [4]. Vitamin D deficiency, more common in individuals avoiding dairy or with limited sun exposure, has been associated with a substantially higher risk of both latent TB infection and progression to active TB [4]. Several studies noted that vitamin D-deficient persons had about a 2-5 fold increased risk of developing active TB [4,10,11]. Although we cannot definitively prove that malnutrition triggered this patient’s TB reactivation, the scenario aligns with the concept that his strict diet-induced immunosuppression opened the door for latent TB to resurface and disseminate. The literature even documents cases of otherwise healthy individuals developing severe TB after extreme diets; for instance, a 2019 report described a patient who developed miliary TB in the context of malnutrition from a long-term vegan diet [12]. In our patient, improved nutritional support (vitamin supplementation and a balanced diet) was an integral part of recovery, alongside antimicrobial therapy. This aspect underlines a broader public health message: in TB control, addressing undernutrition is as important as treating the infection, since undernutrition both predisposes to TB and is worsened by TB in a vicious cycle [5].

This case illustrates the importance of a timely, multi-pronged therapeutic approach in managing adrenal crisis secondary to disseminated TB. The patient’s collapse resulted from two interrelated conditions, adrenal insufficiency and systemic TB, each worsening the other. Prompt corticosteroid therapy reversed shock, while anti-tubercular treatment addressed the underlying infection, leading to rapid clinical improvement. Follow-up confirmed therapeutic response: the patient gained 2 kg, symptoms resolved, electrolytes normalised, and sputum cultures converted to negative. A three-month post-discharge ACTH level of 309 pg/mL confirmed persistent primary adrenal insufficiency, justifying long-term hormone replacement. While ACTH was not measured during admission due to prior immunosuppressive therapy, the diagnosis was supported by integrated clinical, biochemical, imaging, and microbiological evidence. A further limitation is the potential delay in presentation due to TB-related stigma in some South Asian communities, underscoring the need for culturally sensitive education.

## Conclusions

This case highlights the diagnostic and therapeutic challenges of managing Addisonian crisis secondary to disseminated TB, particularly when initial presentations mimic septic shock. Prompt recognition of adrenal insufficiency features, such as hyponatraemia, hyperkalaemia, hypoglycaemia, and hyperpigmentation, and timely administration of intravenous hydrocortisone were pivotal in reversing circulatory collapse and preventing further deterioration. Although global TB prevalence is declining, its role in adrenal destruction remains under-recognised in high-income settings, often resulting in delayed diagnosis and treatment.

The case underscores the importance of maintaining a high index of suspicion for disseminated TB in patients presenting with non-specific systemic symptoms and signs of adrenal insufficiency, even when initial microbiological testing is inconclusive. It also demonstrates the clinical value of integrating endocrine, infectious disease, and nutritional perspectives when evaluating atypical shock presentations in high-risk populations. Optimal management requires prompt endocrine stabilisation, targeted anti-tubercular therapy, and comprehensive long-term care including hormone replacement, nutritional support, and patient education. Early recognition and a multidisciplinary approach are essential to improving outcomes and preventing avoidable mortality.
